# Medical Institutions and Twitter: A Novel Tool for Public Communication in Japan

**DOI:** 10.2196/publichealth.4831

**Published:** 2016-05-12

**Authors:** Yuya Sugawara, Hiroto Narimatsu, Atsushi Tsuya, Atsushi Tanaka, Akira Fukao

**Affiliations:** ^1^ Institute for Promotion of Medical Science Research Yamagata University Faculty of Medicine Yamagata Japan; ^2^ Cancer Prevention and Control Division Kanagawa Cancer Center Research Institute Yokohama Japan; ^3^ Department of Public Health Yamagata University Graduate School of Medicine Yamagata Japan; ^4^ Graduate School of Science and Engineering Yamagata University Yonezawa Japan

**Keywords:** social media, Web 2.0, medical education, consultation guidance, advertisement

## Abstract

**Background:**

Twitter is a free social networking and microblogging service on the Internet. Medical professionals and patients have started to use Twitter in medicine. Twitter use by medical institutions can interactively and efficiently provide public health information and education for laypeople.

**Objective:**

This study examined Twitter usage by medical institutions.

**Methods:**

We reviewed all Japanese user accounts in which the names of medical institutions were described in the user’s Twitter profile. We then classified medical institutions’ tweets by content.

**Results:**

We extracted 168 accounts for medical institutions with ≥500 followers. The medical specialties of those accounts were dentistry and oral surgery (n=73), dermatology (n=12), cosmetic surgery (n=10), internal medicine (n=10), ophthalmology (n=6), obstetrics and gynecology (n=5), plastic surgery (n=2), and others (n=50). Of these, 21 accounts tweeted medical knowledge and 45 accounts tweeted guidance about medical practice and consultation hours, including advertisements. In the dentistry and oral surgery accounts, individual behavior or thinking was the most frequent (22/71, 31%) content. On the other hand, consultation including advertisements was the most frequent (14/23, 61%) in cosmetic surgery, plastic surgery, and dermatology.

**Conclusions:**

Some medical specialties used Twitter for disseminating medical knowledge or guidance including advertisements. This indicates that Twitter potentially can be used for various purposes by different medical specialties.

## Introduction

Twitter is a free social networking and microblogging service on the Internet. Twitter has grown rapidly in popularity [1]. In medicine, increasing numbers of medical professionals and patients use Twitter. We first reported that Twitter networks of patients with cancer have developed so they can exchange information on Twitter [2]. Harris et al reported on tweets about diabetes mellitus by a local health department [3]. In addition to cancer, Twitter is used in various fields, such as dental pain surveillance [4], exploring misunderstanding or misuse of antibiotics [5], and monitoring influenza outbreaks [6-8].

Medical professions have started to use Twitter [9]. For example, some physicians or medical teams use Twitter to share medical information with the public [10-16]. Thus, Twitter use by medical institutions might interactively and efficiently provide public health information and education for laypeople. We could speculate that some of the needs and requests of the public cannot be met by typical medical practice. Thus, medical professionals may be able to conduct personalized educational activities. Furthermore, Twitter can contribute to improved satisfaction with health promotion and medical care.

Information about Twitter use by medical institutions is highly limited and the optimal methods of use remain unclear. In this study, we examined Twitter usage by medical institutions and Twitter’s role in public medical information. This should assist medical institutions that use Twitter in making significant changes from noninteractive, 1-way publicity to interactive information exchange, wherein personal opinions, needs, and requests are disseminated and collected in the future.

## Methods

### Identifying Twitter Accounts

We searched Twitter profiles using a method detailed elsewhere [2]. Briefly, we searched every publicly available user profile on Twitter in Japan. The search words were “hospital” and “clinic” in Japanese. We reviewed all user accounts that included the names of medical institutions, aiming specifically at those with ≥500 followers. As in our previous study’s methods, we included only such large “powerful” accounts [9]. The website used for the profile search was the 16 Profile Search beta version for Twitter [17]. The search was conducted on July 15, 2013. We classified the accounts using Twitter profiles. We excluded accounts that probably were not medical institutions. The accounts with medical institutions’ names were categorized as medical institutions. However, in the case of medical staff, we categorized the accounts by professions. The people who had medical treatment in that medical institution were categorized as patients. Then, we classified the extracted medical institution accounts into the following categories: dentistry and oral surgery, cosmetic surgery, plastic surgery, dermatology, other surgery, and internal medicine including pediatrics.

We collected the latest tweets of the top 5 accounts in terms of number of followers for measuring longitudinal changes. We extracted tweets from 2012 to 2014. The website we used for collecting the latest tweets was TwimeMachine [18], which can collect a maximum of 3200 tweets. This search was conducted on November 5, 2015.

We searched the accounts of medical institutions in English-speaking countries for comparison with the accounts of medical institutions in Japan. The search words were “hospital” and “clinic” in English. We extracted the accounts with the top 10 number of followers, reading medical institution profiles. The website used for collecting the accounts of medical institutions in English-speaking countries was followerwonk [19]. The search was conducted on October 30, 2015. We referred to the URL of medical institutions if we could not extract information about the medical institutions from the Twitter profile alone.

### Categorizing Tweets

We classified the medical institution tweets into 6 categories using the 100 most recent tweets. The categories were medical knowledge (category A); consultation guidance (including advertisements and newsletters from medical institutions; category B); suggestions from patients (category C); links to other home pages only (category D); individual behavior or thinking (category E); and tweets with multiple kinds of content (eg, tweets with a URL link to a home page and medical knowledge tweets from the same account, classified by YS, a medical informatics researcher; category F). In the most recent 100 tweets, the most categorized tweets from categories A to E were the account classifications. When categories A to E were almost equal, we classified the accounts as category F. The categories for tweets that the primary investigator (YS) could not categorize were decided by discussion with the secondary investigator (NH).

### Analyzing Tweet Morphemes

Tweet morphemes in Japanese were analyzed using the method previously developed for frequency comparison of nouns by medical specialties [20]. Briefly, we collected 200 tweets and used the ChaSen (version 2.1 for Windows, Nara Institute of Science and Technology, Ikoma, Nara, Japan) morphological analysis system. We used the normal ChaSen dictionary in morpheme analysis, extracting only nouns from the tweets. Tweets were obtained January 11, 2014.

## Results

### Identifying Medical Institution Accounts

**Figure 1:**
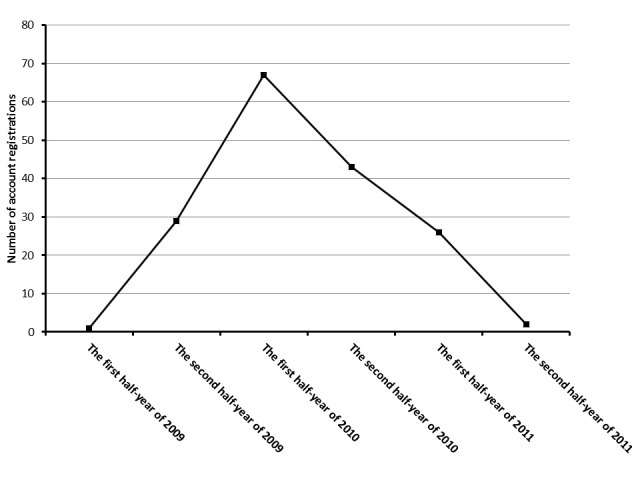
Year of Twitter registration by 168 medical institutions, showing the number of Twitter registrations from 2009 to 2011 per half year. The first registration was in July 2, 2009. The latest registration was on November 27, 2011.

We identified 1211 Twitter accounts with ≥500 followers in which the keywords “hospital” and “clinic” were included in the profile. From those, we extracted 168 accounts by medical institutions using the following keywords in Japanese: *Byoin* (hospital) and *Shinryojo*, *Iin*, or *Kurinikku* (clinic) (Table 1). Among the 168 medical institutions, 30 had registered Twitter accounts in 2009, 110 in 2010, and 28 in 2011 (Figure 1). As of July 15, 2013, the median duration from the day Twitter accounts were registered to the last tweeted day was 1151 (range 596–1526 days).

**Table 1:** Categorized Twitter accounts (relevant Twitter accounts with ≥500 followers on July 15, 2013).

Type of Twitter account		Search words				Total no. of accounts
		*Byoin* (hospital)	*Shinryojo* (clinic)	*Lin* (clinic)	*Kurinikku* (clinic)	
**Accounts related to medicine**						
	Medical institutions	30	6	67	65	168
	Pharmacies	2	0	0	0	2
	Doctors, dentists	86	12	39	53	190
	Nurses, midwives	35	1	0	6	42
	Paramedics (except nurses)	71	1	10	5	87
	Other hospital personnel	68	4	3	7	82
	Tweets about medical knowledge^a^	18	0	6	5	29
	Patients	11	0	1	1	13
	Total	321	24	126	142	613
Accounts unrelated to medicine		356	31	58	153	598
Total no. of accounts		677	55	184	295	1211

^a^The accounts’ proprietors were unknown.

**Figure 2:**
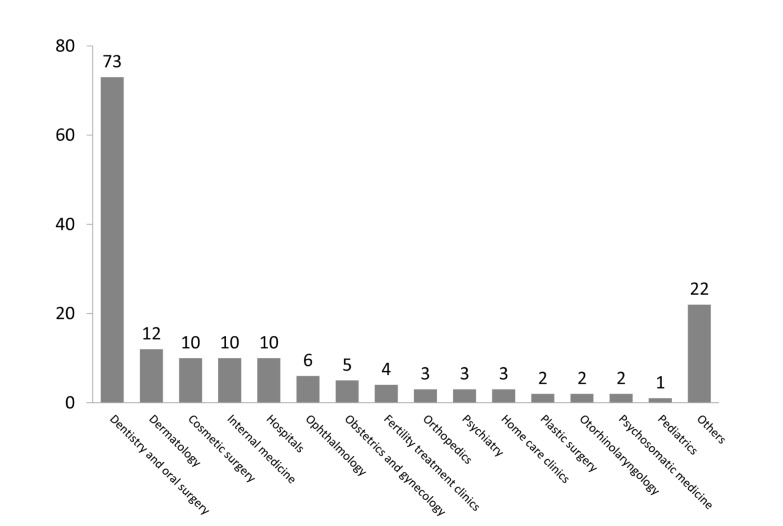
Number of Twitter accounts in each medical specialty. The medical specialties of general hospitals were included in the specialty accounts and not in “hospitals”.

The medical specialties of those accounts were dentistry and oral surgery (n=73), dermatology (n=12), cosmetic surgery (n=10), internal medicine (n=10), ophthalmology (n=6), obstetrics and gynecology (n=5), plastic surgery (n=2), and others (n=50; Figure 2).

We identified 190 Twitter accounts of doctors or dentists in this search, including 10 pediatricians. We searched “pediatrician” using 16 Profile Search beta version for Twitter on March 12, 2014 and identified 12 pediatrician accounts with ≥500 followers.

Table 2 shows the accounts with the top 10 number of followers in Japanese medical institutions. The medical specialties in the accounts having the top 10 followers were dentistry (n=3), cosmetic surgery (n=2), ophthalmology (n=1), therapeutic radiology (n=1), obstetrics and gynecology (n=1), dermatology (n=1), and internal medicine (n=1). Account 1 had the highest number of followers (n=169,407). There was a median of 3129.5 tweets, 29,865 followers, and 3.00 tweets per day.

Table 3 shows the top 10 accounts of medical institutions by number of followers in English-speaking countries. Those accounts were from 6 general hospitals, 3 children’s hospitals, and 1 cosmetic surgery clinic. There was a median of 9072 tweets, 213,314.5 followers, and 3.61 tweets per day.

**Table 2:** The top 10 accounts by number of followers of Japanese medical institutions.

No.	Account name	Hospital or clinic	Medical specialty	Prefecture	Date joined	Total tweets	Followers	Tweets/day^a^
1	@team_nakagawa	Hospital	General hospital (therapeutic radiology)	Tokyo	March 15, 2011	285	169,407	0.33
2	@Sanhujinka	Clinic	Obstetrics and gynecology	Kanagawa	April 7, 2010	81,753	90,906	68.41
3	@biyoudoctor	Clinic	Cosmetic surgery	Tokyo	September 20, 2010	4603	48,151	4.47
4	@SBCLASIK	Clinic	Ophthalmology	Tokyo	March 8, 2010	6303	45,063	5.15
5	@sbcmatsuoka	Clinic	Dermatology	Tokyo	September 24, 2010	1559	32,609	1.52
6	@GVBDO	Clinic	Dentistry	Gifu	May 21, 2010	31,568	27,121	27.43
7	@ryouki4181	Clinic	Dentistry	Osaka	July 6, 2010	1422	21,365	1.29
8	@suzuki1855	Clinic	Dentistry	Yamagata	November 9, 2009	1656	18,914	1.23
9	@Drponchi	Clinic	Internal medicine	Kanagawa	May 19, 2011	7370	16,852	9.35
10	@moriyukabc	Clinic	Cosmetic surgery	Mie	December 5, 2010	359	13,916	0.38
Median						3129.5	29,865	3.00

^a^Up to July 15, 2013.

**Table 3:** The top 10 accounts by number of followers of medical institutions in English-speaking countries.

No.	Account name	Hospital or clinic	Medical specialty	Country	Date joined	Total tweets	Followers	Tweets/day^a^
1	@MayoClinic	Hospital	General hospital	US	April 30, 2008	25,859	1,233,111	9.44
2	@HarvardHealth	Hospital	General hospital	US	March 4, 2009	2,898	998,726	1.19
3	@ClevelandClinic	Hospital	General hospital	US	March 14, 2009	27,644	542,697	11.42
4	@StJude	Hospital	Children’s hospital	US	October 23, 2007	6,567	388,754	2.24
5	@HopkinsMedicine	Hospital	General hospital	US	February 7, 2009	11,469	329,343	4.67
6	@FUEHairClinics	Clinic	Cosmetic surgery	UK	August 10, 2013	738	97,286	0.91
7	@GreatOrmondSt	Hospital	Children’s hospital	UK	October 22, 2009	20,703	82,405	9.41
8	@HospitalsApollo	Hospital	General hospital	India	October 25, 2010	26,494	77,120	14.47
9	@seattlechildren	Hospital	Children’s hospital	US	September 4, 2008	6,675	55,531	2.56
10	@NIHClinicalCntr	Hospital	General hospital	US	February 6, 2009	3,111	52,221	1.27
Median						9072	213,314.5	3.61

^a^Up to October 30, 2015.

**Figure 3:**
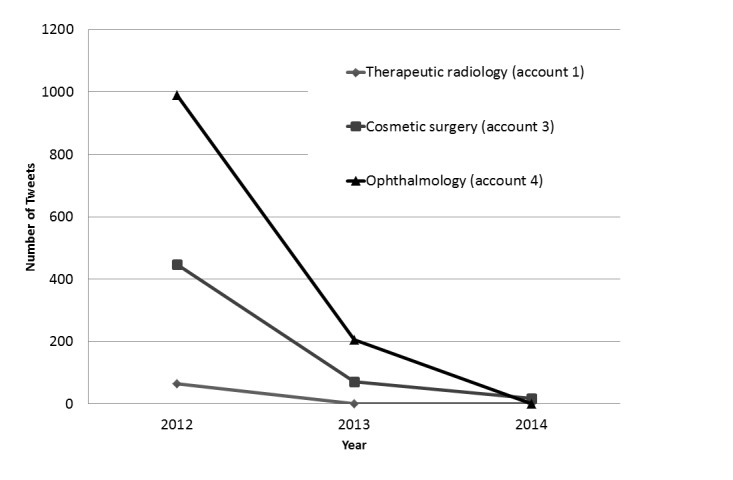
Number of tweets from medical institutions in Japan from 2012 to 2014 with content on therapeutic radiology (account 1), cosmetic surgery (account 3), and ophthalmology (account 4).

Figure 3 shows the top 5 accounts of medical institutions by number of followers in Japan. The therapeutic radiology account (account 1), with the most followers, tweeted 64 tweets in 2012 and had no tweets from 2013 to 2014. We could not collect tweets from an obstetrics and gynecology account (account 2) and a dermatology account (account 5) from 2012 to 2014 due to technology issues. Account 2 had more than 3200 tweets, none of which could be technically collected due to the large numbers of tweets from 2012 to 2014. We could not access account 5 on November 5, 2015.

Figure 3 shows the number of tweets by therapeutic radiology (account 1), cosmetic surgery (account 3), and ophthalmology (account 4) accounts (Table 2) from 2012 to 2014. All tweets from account 1 in 2012 were about the Fukushima nuclear accident and radiation exposure. Account 3 had 77 tweets with links to preoperative and postoperative photographs of all 536 tweets from 2012 to 2014 (14.4%). Account 4 was a clinic that performed LASIK eye surgery. The content of its tweets recommended LASIK surgery, provided information about appointment status in that clinic, and contained advertising. Over 3 years, 80.33% (960/1195) of their tweets contained their phone number.

### Categorizing Tweets

**Figure 4:**
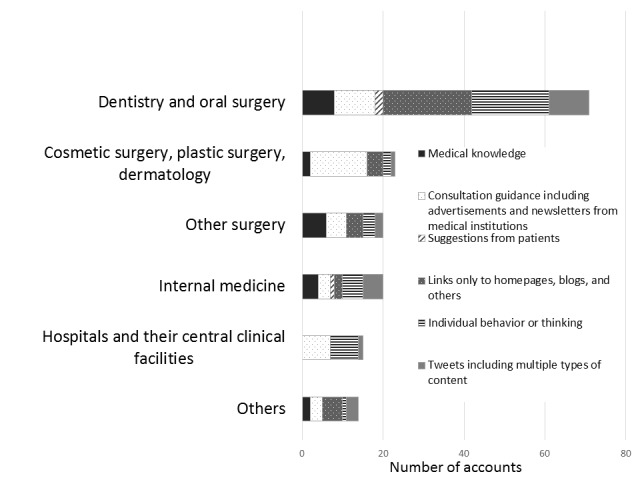
Number of accounts by medical specialty tweeting content categorized as follows: medical knowledge; consultation guidance including advertisements and newsletters from medical institutions; suggestions from patients; links only to homepages, blogs, and others; individual behavior or thinking; and tweets including multiple types of content.

We classified tweets by medical institutions into 6 categories by content (see Table 4). Tweets about consultation guidance including advertisements and newsletters from medical institutions (category B) were most frequent. Figure 4 shows content classification of tweets in each specialty. In cosmetic surgery, plastic surgery, and dermatology, category B was the most frequent (n=14, 61%). In dentistry and oral surgery, category E was the most frequent (n=22, 31%). The accounts of 2 medical institutions were attempting to recruit dentists, dental hygienists, nurses, and others. Table 5 shows sample tweets.

**Table 4:** Number of tweets by categories of contents.

Category	Content	No. of tweets^a^
A	Medical knowledge	21
B	Consultation guidance, including advertisements and newsletters from medical institutions	45
C	Patients’ suggestions	3
D	Links only to home pages, blogs, etc	37
E	Individual behavior or thinking	37
F	Tweets with multiple types of content	20

^a^A total of 5 accounts could not be accessed to obtain tweets on January 11, 2014.

**Table 5:** Sample tweets by content category.

Category	Account no.	Tweet sample^a^
A	1	Internal exposure to radioactive cesium through foods reminds us of “Minamata disease” caused by concentrated organic mercury in the food chain from Chisso Minamata factory. Those who ate fish polluted by organic mercury in high densities accumulated “liposoluble” organic mercury in their brain and frequently experienced neuropathy.
A		On the other hand, radioactive cesium seldom causes biological concentrations like organic mercury. Cesium is an alkali metal close to potassium. Taken into the body, cesium distributes evenly to all cells and is excreted in urine. This was established by analysis of euthanized domestic animals in Fukushima prefecture.
A		Cesium is excreted in urine like potassium. The amount of cesium in the body is halved at nine days in infants and at three months in adults. Unlike organic mercury, cesium is not accumulated in the body. Even when cesium is excreted, it exists on Earth because the half-life of cesium-137 is approximately 30 years.
A	2	Defining blood–brain barrier (BBB) disruption as a hyperdense lesion on the CT scan after endovascular therapy in acute ischemic stroke patients, BBB disruption is independently associated with lower rates of early major neurologic improvement, higher rates of mortality, and hemorrhagic complications. http://****
A	3	We call it a wedge-shaped defect when the sides of the teeth are sharpened. Clenching one’s teeth is the most common cause of wedge-shaped defects. Images→http://****
B	4	****clinic for liposuction and fat infusion! ****clinic is an expert in liposuction with more than 30 thousand cases. We have been chosen for techniques and trust.
B	5	We will start flu shots on October 15. We are accepting reservations from companies for flu shots. Call ** in charge.
C	6	[In patient's voice]: “I found this dentist. This dentist described the treatment I wanted and how to brush well. This dentist explained other things besides treatment, too. If I had brushed my teeth earlier, I would have better teeth. I regret that now.”
D	7	Our blog was renewed. [Report of cardiology training] at http://****
E	8	Let’s drink water because it’s hot.
E	9	I feel that it’s the end of the year due to increasing numbers of patients with hand injuries.
		I feel that it’s spring because of the number of infectious atheroma cases.
F	10	Anticancer activity of extract from grape seeds: Many studies suggest that an extract from grape seeds has anticancer properties. http://****
		Watching and doing dance performances are fun, even if we cannot do them. Everyone says, “If I was XX years younger, I would dance.” Let’s go dancing! Throw away your assumptions and prejudices.

^a^Japanese conversations were translated into English. The number of characters could be more than 140 because the tweets were translated from Japanese sentences.

### Analyzing Tweet Morphemes

The frequency of nouns per medical specialty is shown in Multimedia Appendix 1. In all specialties, words about doctor visits frequently appeared, such as “consultation,” “appointment,” “reception,” and “closed,” (n=23,919). Keywords unique to each specialty appeared frequently (Multimedia Appendix 1). Examples include “decayed tooth” in dentistry, “depilation” in cosmetic surgery, “surgical operation” in other surgery, and “influenza” in internal medicine. Advertisement words such as “campaign” and “inquiry” frequently appeared in cosmetic surgery.

## Discussion

### Twitter Use in Medical Institution Management

In this study, we classified Twitter accounts by medical practice, including dentistry and oral surgery, cosmetic surgery, plastic surgery, dermatology, other surgery, and internal medicine including pediatrics, and by tweet contents. Powerful accounts with ≥500 followers varied by clinical specialties, as did Twitter use indicators such as amount of active Twitter use. There were 179,857 medical institutions in Japan [21]. We identified 168 medical institution Twitter accounts with ≥500 followers. These accounted for 0.09% of all medical institutions in Japan. There were more dentistry and cosmetic surgery accounts, whereas there were fewer in internal medicine, surgery, and pediatrics (Figure 2). This result may have been affected by differing medical institution management by specialty and the Japanese medical insurance system. In Japan, medical costs are covered by universal insurance. People pay 10%-30% of medical care costs on their own [22-24]. Most medical practice is conducted under health insurance. However, some medical practices in cosmetic surgery and dentistry are not covered by health insurance; indeed, most practices in cosmetic surgery are not covered by insurance. Infertility and obstetric care are also not covered unless complications are diagnosed. Patients must pay the full amount of the medical expenses not covered by health insurance, and medical institutions can freely set the cost of uncovered practices. Therefore, improving brand images to maintain higher pricing of medical costs is of much greater financial interest to medical institutions conducting uncovered practices than it is to those conducting primarily covered practices. This suggests that the management of a medical institution affects the number of Twitter accounts and the contents of medical institution tweets. Future studies will allow appropriate interpretation.

Many of the top 10 Japanese accounts were medical institutions of cosmetic surgery and dental clinics, which frequently performed medical treatment not covered by health insurance. This suggested that the accounts of cosmetic surgery clinics possibly used Twitter for advertising. Few of the accounts were from general hospitals, while many were from clinics. In comparison, many of the top 10 accounts from English-speaking countries were from general hospitals. The British Medical Association has published guidelines about social media use among doctors and medical students in the United Kingdom [25]. However, there are no such guidelines in Japan. Clinics can establish Twitter use policies rapidly and independently. In large general hospitals, the decision to use Twitter may be difficult. In addition, the tweets of some medical institutions decreased annually. The accounts of cosmetic surgery and ophthalmology clinics performing LASIK surgery might not have been attracting customers. In Japan, medical institutions temporarily used Twitter without defined purposes. It is necessary to perform detailed investigations on Twitter use by medical institutions.

### Tweet Contents

Tweet contents were also markedly different between the specialties. For example, cosmetic surgery accounts, where most practices are not covered by insurance, tended to tweet consultation guidance, including advertisements. We also found advertisement tweets covering introductions to clinic operations, consultation hours, and working hours of the doctor in charge of operations. Of the tweets by cosmetic surgery accounts, 38% (9/24) were categorized as advertisements, defined as tweets with concrete prices or discounts. Most practices in cosmetic surgery are not covered by insurance and it is necessary to attract customers or collect patients. Thus, Twitter might be used as an advertising medium to attract cosmetic surgery patients. This supported our inference that medical institution management affected Twitter use.

On the other hand, the Twitter accounts of some specialties were used for medical knowledge and education. Words about pregnancy frequently appeared in the other surgery category, such as “uterus,” “spermatozoon,” and “infertility” (Multimedia Appendix 1). These Twitter accounts probably disseminated medical knowledge about pregnancy or infertility. There were 4 infertility treatment clinic accounts. In fact, 3 infertility treatment clinics tweeted frequently about infertility, 2 of them focusing on education for men. One of the infertility treatment clinics’ account was deleted because it could not be accessed for tweets, even though it specified disseminating medical knowledge about infertility in its Twitter profile. This is one example of medical knowledge education. In this area, Twitter might be a useful tool for disseminating medical knowledge. Pregnant women often use the Internet as an information source [26-28]. Obstetricians and gynecologists, midwives, nurses, and others may also educate pregnant women through Twitter.

Some Twitter accounts used Twitter for both attracting patients and disseminating medical knowledge. Words about dental practice frequently appeared in the dentistry and oral surgery accounts, such as “decayed tooth,” “implant” (*inpuranto* in Japanese), “orthodontic,” and “prophylaxis.” This indicated that dentistry and oral surgery accounts frequently tweeted about dental practice. Implants and most orthodontic care are not covered by health insurance; thus, those words about dental practice might frequently appear not only to disseminate medical knowledge about implants and orthodontic care but also to attract customers. Moreover, patient home prevention is the most important practice in preventive dentistry. Thus, dentistry and oral surgery accounts might disseminate information about preventive dentistry for patients with figures and photographs through Web links. In fact, the 57 dentistry and oral surgery accounts in this study had a Web link in their tweets. Because Twitter users frequently wish to share Web content and tweets are restricted to 140 characters in length, tweets frequently shortened URLs [29]. The details of medical information were explained through Web links in Twitter. Medical institutions might effectively disseminate medical information using Twitter with Web links in every specialty.

### Use of Twitter in Internal Medicine, Pediatrics, and Surgery

In some specialties, such as internal medicine, surgery, and pediatrics, Twitter was seldom used. Of the 168 accounts, 83 (49.4%) were dentistry and cosmetic surgery accounts. In contrast, only 11 accounts (6.5%) were in internal medicine, pediatrics, and surgery. In those specialties, most medical practices in Japan are covered by medical insurance. Thus, the institutions likely do not need to attract customers or collect patients. In particular, there was only 1 pediatric Twitter account, which was significantly lower than in other specialties. Parents with small children often use Twitter because they have high Internet literacy [30,31]. We hypothesized that disseminating medical knowledge about pediatric practice to parents would be useful, but our study did not support this hypothesis. However, 3 children’s hospitals were included in the top 10 accounts by number of followers in English-speaking countries. Disseminating medical knowledge about pediatric practice to parents would be useful in English-speaking countries. We should investigate what medical institutions in English-speaking countries tweeted to demonstrate our hypothesis. We searched Twitter accounts for pediatricians a second time because of the lack of pediatric clinic accounts on Twitter in the first round of the study. We found 10 pediatricians among 190 doctor or dentist Twitter accounts, but 21 pediatrician accounts with ≥500 followers. We do not know the reasons for this inconsistency in the number of pediatric clinic and pediatrician accounts. However, first, it is possible that, in pediatrics, social media usability is not widely known. Second, other social media besides Twitter may have been more commonly used. It may be necessary to perform specific investigations on pediatricians and pediatric clinics. On the other hand, most patients in internal medicine and surgery are older or have a chronic disease. It may be difficult to disseminate medical information via Twitter to such elderly patients who do not use social media or the Internet. For these patients, it is likely that written dissemination of medical information will continue to be important.

### Study Limitations

In this study, we first showed how medical institutions use Twitter and what they tweet. Nevertheless, several issues remain for discussion.

First, we conducted this study with power accounts (having ≥500 followers) as the focus. Thus, we might not have obtained the same results if we had included accounts with fewer followers. About 98% of Twitter accounts have fewer than 500 followers in Japan [32]. It is likely that some smaller accounts contribute to public health via Twitter. Therefore, such accounts should be investigated to provide a broad view of medical institutions’ Twitter use.

Second, we did not examine the medical accuracy of tweets in this study. Several studies have examined deleterious and inaccurate information on websites and social media [33,34]. It will be necessary to investigate tweet medical reliability and accuracy of tweets. Although Twitter is a useful tool for communication, the dissemination of inaccurate medical knowledge by Twitter might negatively affect people. Thus, it is important to examine Twitter’s potential negative impact in future studies.

Third, the results of this study depended on the search site; however, it remains unclear what Web search tools are sufficiently accurate and adequate. Some search sites for Twitter accounts exist, such as twpro, Twitter Search, and Twitter Profile Search [35-37]. Our preliminary search showed differences in search results between search sites (data not shown). We used 16 Profile Search beta version for Twitter for account searches for the following reasons. In this tool, AND, OR, and NOT searches were available. Furthermore, there were many kinds of output data, including account profiles, and number of tweets and followers, and the characteristics of search tools must be investigated in detail in future studies.

Fourth, we did not examine whether Twitter advertisements conformed to guidelines about medical institution advertising. Advertising by medical institutions is regulated by the Japanese Medical Care Act [38]. The Ministry of Health, Labour and Welfare provides guidelines for medical advertisements [39,40]. These detailed guidelines regulate specialties, licenses of specialists, and so on. Descriptions of curative effects, noting the number of times a doctor performed the operation, and showing before-and-after photographs are not permitted. It is also important for patients to know the medical cost. The guidelines state that advertisements emphasizing medical expenses should be regulated. There was a tweet that did not follow this guideline in the study. Thus, Twitter advertisements should be investigated to determine whether they follow medical advertisement guidelines.

### Conclusions

The number of Twitter accounts by medical institutions and the tweet contents differed by medical specialty. Some Twitter accounts attracted customers or collected patients. On the other hand, some accounts provided medical knowledge. Thus, Twitter potentially can be used for various purposes by different medical specialties.

## Appendix

Multimedia Appendix 1 Twenty most frequently tweeted Japanese nouns in each medical specialty.
