# Supplementary material for: Medical Institutions and Twitter: A Novel Tool for Public Communication in Japan
Source: JMIR Public Health Surveill. 2016 May 12;2(1):e19. doi: 10.2196/publichealth.4831 (PMC4869231; doi:10.2196/publichealth.4831)
Supplement: Multimedia Appendix 1 [file publichealth_v2i1e19_app1.pdf]

Multimedia Appendix 1. Twenty most frequently tweeted Japanese nouns in each medical specialty.

Total		Dentistry and oral surgery		Cosmetic surgery, plastic surgery, and dermatology		Internal medicine				Hospitals		Others	
Noun	No.	Noun	No.	Noun	No.	Noun	No.	Noun	No.	Noun	No.	Noun	No.
dentistry	3,643	dentistry	3,621	appointment	551	reception	569	consultation	387	hospital	586	pathology	401
consultation	3,057	teeth	2,948	clinic	449	medical examination	506	renewal	328	renewal	384	medicine	368
teeth	3,055	treatment	1,697	fat	425	clinic	454	medicine	315	consultation	338	clinic	363
treatment	2,721	consultation	1,473	Osaka[a]	394	treatment	377	infection	270	surgical operation	278	diagnosis	264
renewal	2,178	*Iin* (clinic)	1,028	renewal	391	surgical operation	373	clinic	255	nursing	272	Hachioji[a]	234
clinic	1,889	patient	877	consultation	384	checkup	302	information	226	medicine	252	smoking abstention	223
patient	1,578	renewal	788	*Hada* (skin)	379	Shinjuku[a]	298	regular	206	internal medicine	244	renewal	216
medicine	1,318	[Shi]*shu* (periodontal)	644	duty	378	consultation	270	patient	189	doctor	215	consultation	205
appointment	1,194	dentist	622	Umeda[a]	377	male	265	person	188	health	214	checkup	200
reception	1,132	decayed tooth	618	treatment	348	ophthalmology	242	schedule	182	notification	203	health	177
health	1,074	*In*[pranto] (implant)	585	depilation	319	myopic	235	health	176	course	198	mind	157
hospital	1,073	[In]*Pranto* (implant)	534	regular	300	infertility	231	hospital	158	holding	193	medical accounting	153
Iin (clinic)	1,056	orthodontic	472	director	298	cancer	196	Fujita[b]	154	training	189	appointment	126
director	907	health	428	campaign	297	appointment	193	doctor	150	rece	177	*Ken*[s	124

										ption		hin] (health checkup)	
person	874	column	425	*Kuri*[nikku] clinic	293	pregnancy	176	disease	147	breast	172	institution	115
photograph	859	person	420	skin	292	uterus	175	treatment	146	Jonan[a]	167	treatment	113
prophylaxis	853	photograph	409	suction	279	spermatozoon	174	Japan	142	daily issue	167	Yonago[a]	105
[Shi]*shu* (periodontal)	841	director	401	effect	263	patient	169	vaccine	137	outpatient	143	patient	98
medical examination	817	staff	375	inquiry	245	completion	154	influenza	134	closed	142	hospital	97
closed	811	prophylaxis	336	beauty	243	director	151	checkup	134	information	142	prophylaxis	95

[a]: Denotes a city, town, or business district in Japan.

[b]: A family name.
